# Roles of CDKN1B in cancer?

**DOI:** 10.18632/aging.100786

**Published:** 2015-08-20

**Authors:** Barbara Belletti, Gustavo Baldassarre

**Affiliations:** Division of Experimental Oncology 2, Department of Translational Research, C.R.O. Aviano IRCCS, National Cancer Institute, 33081 Aviano, Italy

Breast cancer (BC) is the most common cancer among women, with nearly 1.7 million new cases diagnosed in 2012 worldwide, ranking as the fifth cause of death for cancer (522,000 deaths/y). The wide introduction of molecular analyses allowed the comprehension that BC is not a single entity but should be divided into, at least, 4 major subtypes: Luminal A, Luminal B, HER2 positive and triple negative/basal-like. Luminal Breast Cancer (LBC) represents the most common subtype, accounting for more than 60% of all diagnosed BC. In clinical practice, only few characteristics, such as Estrogen Receptor (ER), Progesteron Receptor (PR), HER2 and Ki67 expression, are currently used to distinguish Luminal A (LBC-A) (ER+ and/or PR+, HER2−, low Ki67) and Luminal B (LBC-B) (ER+ and/or PR+ HER2− or HER2+, high Ki67) [1]. However, this oversimplified classification has profound therapeutic implication, since based on the relative expression of these markers, LBC patients will or will not receive hormone-, chemo- or targeted-therapies [1]. When compared with LBC-A, LBC-B displays higher rate of early recurrences and worse prognosis and represents a subgroup for which the choice of the optimal therapy still represents a difficult task for the clinician [1]. In fact, clear biomarkers to select the most appropriate (hormone- with or w/out chemo-) therapy in LBC-B, still have to be validated and introduced to the clinic [1].

Recently, next generation sequences (NGS) analyses have further contributed to ameliorating the molecular classification of BC, identifying potential driver mutations. These mutations are in part common to the different BC subtypes and in part specific for each subtype. Among the latter, the tumor suppressor gene CDKN1B, encoding for the cell cycle inhibitor p27^Kip1^, has been identified as a driver gene, mutated exclusively in LBC [2]. Interestingly, expression and functional studies demonstrated that high p27^Kip1^ expression predicts sensitivity to endocrine and chemotherapy in LBC patients, while p27^Kip1^ downregulation predicts resistance to radiotherapy and anti-HER2 therapies.

So far, CDKN1B was found mutated only in few other neoplasia, including prostate cancer (PC) and small intestine neuroendocrine tumors (SI-NETs), a rare carcinoma arising from endocrine precursor cells, in which CDKN1B represents the most frequently mutated gene [3]. CDKN1B germline mutations have been also proposed to be the cause of Multiple Endocrine Neoplasia type 4 (MEN4), an autosomal dominant disorder, characterized by the occurrence of tumors in endocrine glands [4]. These data, not only confirm that CDKN1B is a *bona fide* tumor suppressor gene in humans, as widely demonstrated in mice, but also suggest that p27^Kip1^ deregulation is particularly involved in endocrine tumors and, more generally, in cancers that rely on or respond to hormones, such as LBC.

It is worth noting that most of the described CDKN1B mutations in carcinomas (LBC, SI-NET and PC) are nonsense and/or small deletion/insertion that cause the production of a truncated protein that could retain or not the cyclin-CDK binding domain but invariably lacks the C-terminal portion [2,3]. Conversely, MEN4 adenomas usually harbor CDKN1B missense mutations, with no impact on p27^Kip1^ C-Terminus [4].

This observation raises the possibility that the C-terminal portion of p27 has a particularly relevant role in the onset/development of human endocrine-related carcinomas. Accordingly, it is widely reported that the C-terminus is responsible for many CDK-independent functions of p27^Kip1^, such as transcription and regulation of actin- and microtubule (MT)-cytoskeleton, impacting on cell differentiation, growth, motility and cytokinesis [5,6].

In this context, our lab have demonstrated that the last 28 aminoacid of p27^Kip1^ are necessary for its binding to the MT-destabilizing protein stathmin [5] and that this interaction plays relevant roles *in vivo*, contributing to the regulation of cell proliferation and to tumor onset [7]. Interestingly, a p27^E171^* mutant, losing the last 28 aminoacids, has been identified as driver mutation in LBC [2]. Moreover, we recently proved that p27 also acts as RNA binding protein able to modulate miR-233 availability eventually regulating cell proliferation in response to contact inhibition [6]. This property is lost by the p27^K134fs^ mutant similarly identified as driver mutation in LBC [2,6].

Based on these and other emerging data we can anticipate that other aspect of CDKN1B functions will emerge and will help in clarifying its roles in LBC (and other carcinomas) onset, progression and response to therapies. To reach this aim we absolutely need to better delineate its function using the appropriate *in vitro* and *in vivo* models with a mind as open as possible.

## References

[1] Ades F (2014). J Clin Oncol.

[2] Belletti B, Baldassarre G (2012). Cell Cycle Georget Tex.

[3] Francis JM (2013). Nat Genet.

[4] Lee M, Pellegata NS (2013). Front Horm Res.

[5] Baldassarre G (2005). Cancer Cell.

[6] Armenia J (2014). Oncotarget.

[7] Berton S (2014). Cell Cycle Georget Tex.

